# Brentuximab vedotin consolidation after autologous stem cell transplantation for Hodgkin lymphoma: A Fondazione Italiana Linfomi real‐life experience

**DOI:** 10.1002/hon.2939

**Published:** 2021-11-01

**Authors:** Fulvio Massaro, Vincenzo Pavone, Piero Maria Stefani, Barbara Botto, Alessandro Pulsoni, Caterina Patti, Maria Cantonetti, Andrea Visentin, Potito Rosario Scalzulli, Andrea Rossi, Sara Galimberti, Michele Cimminiello, Guido Gini, Maurizio Musso, Marco Sorio, Annalisa Arcari, Vittorio Ruggero Zilioli, Mario Luppi, Donato Mannina, Alberto Fabbri, Giuseppe Pietrantuono, Ombretta Annibali, Agostino Tafuri, Eleonora Prete, Antonino Mulè, Elisa Barbolini, Luigi Marcheselli, Stefano Luminari, Francesco Merli

**Affiliations:** ^1^ PhD Program in Clinical and Experimental Medicine University of Modena and Reggio Emilia Modena Italy; ^2^ Hematology Unit Azienda Unità Sanitaria Locale‐IRCCS di Reggio Emilia Reggio Emilia Italy; ^3^ Department of Hematology and Bone Marrow Transplant Hospital Card. G. Panico Tricase Italy; ^4^ Hematology Unit General Hospital Ca' Foncello Treviso Italy; ^5^ Division of Hematology Città della Salute e della Scienza Hospital and University Torino Italy; ^6^ Department of Translational and Precision Medicine Sapienza University Rome Italy; ^7^ Division of Onco‐Hematology Azienda Villa Sofia‐Cervello Palermo Italy; ^8^ Unit of Lymphoproliferative Disorders Policlinico Tor Vergata Rome Italy; ^9^ Hematology and Clinical Immunology Unit Department of Medicine (DIMED) University of Padua Padua Italy; ^10^ Department of Hematology Casa Sollievo della Sofferenza San Giovanni Rotondo Italy; ^11^ Hematology Azienda Socio Sanitaria Territoriale Papa Giovanni XXIII Bergamo Italy; ^12^ Division of Hematology Department of Clinical and Experimental Medicine University of Pisa Italy; ^13^ Hematology San Carlo Hospital Potenza Italy; ^14^ Division of Hematology Azienda Ospedaliera Universitaria Ospedali Riuniti Ancona Italy; ^15^ Department of Oncology Hematology and BMT Unit Casa di Cura La Maddalena Palermo Italy; ^16^ Department of Clinical and Experimental Medicine Hematology and Bone Marrow Transplant Unit University of Verona Verona Italy; ^17^ Hematology Unit Ospedale Guglielmo da Saliceto Piacenza Italy; ^18^ Division of Hematology ASST Grande Ospedale Metropolitano Niguarda Milano Italy; ^19^ Department of Medical and Surgical Sciences University of Modena and Reggio Emilia Modena Italy; ^20^ Unit of Haematology Azienda Ospedaliera Papardo Messina Italy; ^21^ Hematology Azienda Ospedaliero‐Universitaria Senese Siena Italy; ^22^ Hematology and Stem Cell Transplantation Unit IRCCS Centro di Riferimento Oncologico della Basilicata Rionero in Vulture Italy; ^23^ Unit of Haematology and Stem Cell Transplantation Campus Bio‐Medico University Rome Italy; ^24^ Department of Clinical and Molecular Medicine and Hematology Sant’Andrea – University Hospital – Sapienza University of Rome Rome Italy; ^25^ Gruppo Amici dell'Ematologia GRADE‐Onlus Foundation Reggio Emilia Italy; ^26^ Fondazione Italiana Linfomi Onlus Modena Italy

**Keywords:** AETHERA trial, autologous stem cell transplantation, brentuximab vedotin, hodgkin lymphoma

## Abstract

The standard management for relapsed or refractory classical Hodgkin lymphoma (cHL) is salvage therapy followed by autologous stem cell transplantation (ASCT). This strategy allows almost 50% of patients to be cured. Post‐ASCT maintenance treatment with brentuximab vedotin (BV) confers improved progression‐free survival (PFS) to cHL patients at high risk of relapse. We investigated the outcome of 105 cHL patients receiving post‐ASCT BV maintenance in the real‐life setting of 23 Italian hematology centers. This population included naïve patients and those previously exposed to BV. Median follow‐up was 20 months. Patients presented a median of two lines of treatment pre‐ASCT, with 51% receiving BV. Twenty‐nine percent of patients had at least two high‐risk factors (refractory disease, complete response [CR] less than 12 months, extranodal disease at relapse), while 16% presented none. At PET‐CT, a Deauville score (DS) of 1–3 was reported in 75% and 78% of pre‐ and post‐ASCT evaluations, respectively. Grade 3–4 adverse events (AEs), mainly peripheral neuropathy, were observed in 16% of patients. Three‐year PFS and overall survival (OS) were 62% and 86%, respectively. According to BV exposure, 3‐year PFS and OS were 54% and 71%, respectively, for naïve and 77% and 96%, respectively, for previously exposed patients. Refractory disease (hazard ratio [HR] 4.46; *p* = 0.003) and post‐ASCT DS 4–5 (HR 3.14; *p* = 0.005) were the only two factors significantly associated with PFS reduction in multivariable analysis. Post‐ASCT BV maintenance is an effective, safe treatment option for cHL naïve patients and those previously exposed to BV.

## 1 INTRODUCTION

Classical Hodgkin lymphoma (cHL) is a highly curable disease, with complete remission (CR) rates of 75%–90% after standard first line treatment.^1^, ^2^ However, for the non‐negligible proportion of patients presenting relapsed or refractory disease, the best treatment option is salvage therapy, followed by consolidation with autologous hematopoietic stem cell transplantation (ASCT),^3^ which cures almost 50% of these patients.^4^, ^5^ Several studies have analyzed the risk factors associated with poor outcomes in this setting, finding that primary refractory disease, CR duration of less than 12 months, and extranodal disease at relapse were related to reduced progression‐free survival (PFS) rates.^6^, ^7^, ^8^ More recently, the predictive role of positron emission tomography (PET)‐computed tomography (CT) has been validated, with worse outcomes reported for patients presenting metabolically active disease before ASCT.^9^ During past years, several attempts have been done to change standard strategy and improve the outcome for these patients, unsuccessfully.^10^, ^11^, ^12^, ^13^

Brentuximab vedotin (BV) is an anti‐CD30 monoclonal antibody, initially approved for cHL patients with progressive disease after ASCT. A phase 2 trial found that, when used as a single agent, BV determined an overall response rate (ORR) and a CR rate of 75% and 34%, respectively.^14^ In the randomized phase 3 AETHERA trial, cHL patients at high risk of progression or relapse after ASCT (at least one of the following criteria: primary refractory disease, CR < 12 months, extranodal disease at relapse) were randomized to receive either consolidation treatment with BV or placebo. BV consolidation treatment was associated with a significant reduction in the risk of progression compared to placebo, with 5‐year PFS rates of 59% and 41%, respectively, leading to BV's approval in this setting.^15^, ^16^ There are only limited real‐word data on the use of BV as post‐ASCT consolidation treatment: the French AMAHRELIS study, presented at the 2020 American Society of Hematology (ASH) congress, and a recently published Turkish experience.^17^, ^18^ It must be underlined that the AETHERA trial excluded patients who had previously received BV. Based on the data emerging on BV efficacy in the salvage setting, both as a single agent and in combination with chemotherapy, an increasing number of patients now receive BV before ASCT.^19^, ^20^, ^21^, ^22^ However, an issue to be clarified concerns toxicity, and particularly peripheral neuropathy, which was observed in 67% of the patients in the AETHERA trial, leading to treatment discontinuation in 23% of cases.^15^

We report here the results of a multicenter real‐life retrospective study on 105 cHL patients treated with BV as consolidation after ASCT.

## 2 MATERIALS AND METHODS

This was a multicenter retrospective study by the Fondazione Italiana Linfomi (FIL) on patients with relapsed or refractory cHL treated with BV in 23 Italian centers between April 2011 and August 2020. Patients were eligible if they had received at least two cycles of BV after ASCT, regardless of prior lines of treatment. The following data were collected at diagnosis and relapse by the treating physician from hospital records: age, sex, prognostic scores (EORTC score for limited‐stage disease, IPS for advanced‐stage disease), B symptoms (fever, night sweats, and weight loss of more than 10% of body mass in the previous 6 months), Ann Arbor stage and risk factors for poor PFS (refractory disease, CR < 12 months, extranodal disease at relapse), and total number of lines of treatment prior to ASCT. Data on disease response from PET‐CT or CT alone were collected after each line of treatment, and both before and after ASCT. For BV consolidation treatment, we collected the following data: the number of cycles administered, any dose reduction, and any adverse event (AE) causing premature interruption or discontinuation when applicable, including allergic reactions, infections, peripheral neuropathy, liver toxicity, and fatigue. Data concerning the type of treatment for relapse after ASCT were recorded, even when the patient proceeded to allogeneic SCT (allo‐SCT). Patient follow‐up was censored at the most recent hospital visit or death. The data were locked and analyzed in September 2020.

As per approved treatment label in Italy, BV had to be administered at 1.8 mg/kg once every 3 weeks for up to 16 doses.

The primary aim of this study was to determine the PFS taking as reference data reported in the AETHERA trial, using data from the placebo arm of that study to assess the benefit deriving from BV consolidation strategy.

The primary study endpoint was PFS, which was calculated from the initiation of BV after ASCT to the time of relapse, disease progression, or death, whichever occurred first. The secondary endpoints were OS, overall response (OR) and CR, AE rates. OS was calculated from the initiation of BV after ASCT according to validated criteria.^23^ Response rates were defined by the treating physician based on either PET‐CT or standard CT. AE grades are reported according to the Common Toxicity Criteria for Adverse Events (CTCAE) Version 5.0. We recorded the therapeutic approach to peripheral neuropathy and the AE grade after the specific treatment.

Continuous covariates are summarized with the median and range, categorical covariates as absolute value and percent proportions. PFS and OS were calculated using the Kaplan–Meier method. A Cox proportional hazards model was used to estimate the HR and its confidence interval at 95% (95% CI). Univariable and multivariable analyses were carried out by means of Cox proportional hazards regression. All tests were two‐sided.

## 3 RESULTS

We included 105 patients in this retrospective analysis, with a median follow‐up time of 20 months (range 2–108). Baseline features are summarized in Table 1. Patients received a median of two lines of treatment before ASCT. The most commonly used salvage therapies were IGEV (ifosfamide, gemcitabine, vinorelbine) (no. 45; 41% and 3% of all first‐ and second‐line salvage treatments, respectively), BEGEV (bendamustine, gemcitabine, vinorelbine) (no. 27; 26% and 2%, respectively), DHAOX (dexamethasone, cytarabine, oxaliplatin) (no. 24; 12% and 19%, respectively) and DHAP (dexamethasone, cytarabine, cisplatin) (no. 12; 8% and 6%, respectively).

**TABLE 1:** Baseline characteristics of patients (*n* = 105)

Characteristics		Patients, no.	Missing, no.
Median age, years (range)		33 (18–68)	
Sex, male (%)		59 (56%)	
Time from diagnosis to BV, months (range)		21 (11–114)	
First‐line treatment	ABVD	96 (91%)	
	BEACOPP esc	5 (5%)	
	CHOP	4 (4%)	
Number of salvage line therapies pre‐ASCT	1	48 (46%)	
	2	42 (40%)	
	3+	15 (14%)	
First salvage therapy	IGEV	43 (41%)	
	BEGEV	26 (25%)	
	DHAOX	12 (11%)	
	DHAP	8 (8%)	
	BV	4 (4%)	
	Other Bendamustine‐based	4 (4%)	
	Other	7 (7%)	
Second salvage therapy	BV	38 (61%)	
	DHAOX	12 (19%)	
	DHAP	4 (6%)	
	IGEV	2 (3%)	
	BEGEV	1 (2%)	
	ICE	1 (2%)	
	Other Bendamustine‐based	1 (2%)	
	Other	3 (5%)	
Refractory disease	Yes	50 (48%)	
Duration remission <12 months	Yes	51 (49%)	
Extranodal involvement at relapse	Yes	23 (22%)	
Advanced stage at relapse	Yes	46 (44%)	
PET‐CT pre‐ASCT, DS	1–3	72 (75%)	4 (4%)
	4–5	24 (25%)	
PET‐CT post‐ASCT, DS	1–3	68 (78%)	18 (17%)
	4–5	19 (22%)	
Pre‐ASCT BV treatment	Yes	54 (51%)	

Abbreviations: ABVD, doxorubicin, bleomycin, vinblastine, dacarbazine; ASCT, autologous stem cell transplantation; BEACOPP esc, doxorubicin, cyclophosphamide, etoposide, procarbazine, prednisolone, bleomycin, vincristine; BEGEV, bendamustine, gemcitabine, vinorelbine; BV, brentuximab vedotin; CHOP, cyclophosphamide, doxorubicin, vincristine, prednisolone; DHAOX, dexamethasone, cytarabine, oxaliplatin; DHAP, dexamethasone, cytarabine, oxaliplatin; DS, Deauville score; ICE, ifosfamide, carboplatin, etoposide; IGEV, ifosfamide, gemcitabine, vinorelbine.

Twenty‐nine (28%) patients received radiotherapy before ASCT: 22 (21%) as programmed treatment while seven (7%) as consolidation on residual disease.

Fifty‐one percent (54 patients) of the total population also received BV before ASCT: in this setting, BV was most frequently employed as second salvage treatment (38; 70%). The median number of pre‐ASCT BV cycles was four (range 2–11). Among the pre‐ASCT high‐risk factors, 30 (29%) patients presented at least two factors, while 17 (16%) did not show any. Particularly, 51 (49%) patients presented a CR duration of less than 12 months, 50 (48%), refractory disease, and 23 (22%), extranodal disease at relapse. PET‐CT evaluation before and after ASCT reported a Deauville score (DS) 1–3 in 72 (75%) and 68 (78%) patients, respectively. In the cohort of patients presenting a positive PET‐CT after ASCT (no. 22; 21%), most (68%) did not receive BV as salvage therapy. The only feature significantly associated to DS 4–5 before ASCT was the presence of a refractory disease (*p* = 0.038).

The median time from cHL diagnosis to the start of BV consolidation was 21.2 months (range 8.3–272.4). The median number of BV consolidation cycles was 10. Overall, 56% (59/105) of patients received 16 cycles of BV: 60% of those who were treated with BV pre‐ASCT and 43% of those who were not. Causes for treatment interruption were AE (no. 15; 33%), disease progression (no. 13; 28%), consolidation with allo‐SCT (no. 8; 17%), physician decision (no. 6; 13%), other (no. 1; 2%), or reason not available (no. 3; 6%). Characteristics of BV consolidation are listed in Table 2. Among the grade 3–4 AEs leading to treatment interruption, we recorded eight peripheral neuropathies (PN), four infections, two infusion reactions, and one liver toxicity (Table 3). Median time to discontinuation was 7 months.

**TABLE 2:** Characteristics of consolidation treatment with BV after ASCT

Characteristics		No.	Missing, no. (%)
Median number of cycles (range)		10 (2–16)	–
BV discontinuation	Yes	46 (44%)	–
Cause for discontinuation	AE	15 (33%)	–
	PD	13 (28%)	–
	Allo‐SCT	8 (17%)	–
	Physician decision	6 (13%)	–
	Other	1 (2%)	–
	NA	3 (6%)	–
Reduction dose	Yes	8 (7%)	–
Best PET‐CT response, DS	1–3	73 (86%)	20 (19%)
	4–5	12 (14%)	
Relapse during or after BV consolidation	Yes	30 (29%)	‐

Abbreviations: AE, adverse event; Allo‐SCT, allogeneic stem cell transplantation; ASCT, autologous stem cell transplantation; BV, brentuximab vedotin; DS, Deauville score; NA, not available; PD, progressive disease.

**TABLE 3:** Toxicity during BV consolidation treatment

AE	Grade 1–2	Grade 3	Grade 4	Total
Peripheral neuropathy	14	9	0	23
Infection	1	3	1	5
Fatigue	1	0	0	1
Infusion reaction	0	0	2	2
Hepatic toxicity	1	0	1	2
Other	1	0	0	1
Total	18	12	4	34

Abbreviations: AE, adverse event; BV, brentuximab vedotin.

We reported 23 cases (21%) of PN, 14 (61%) grade 1–2 and 9 (39%) grade 3 events: treatment consisted of the administration of pregabalin in nine cases, the administration of antioxidants in seven cases, and BV dose reduction in two cases. Of these 18 patients who were treated for PN, seven presented a reduction in neuropathy severity and four had a complete resolution of symptoms.

**FIGURE 1:**
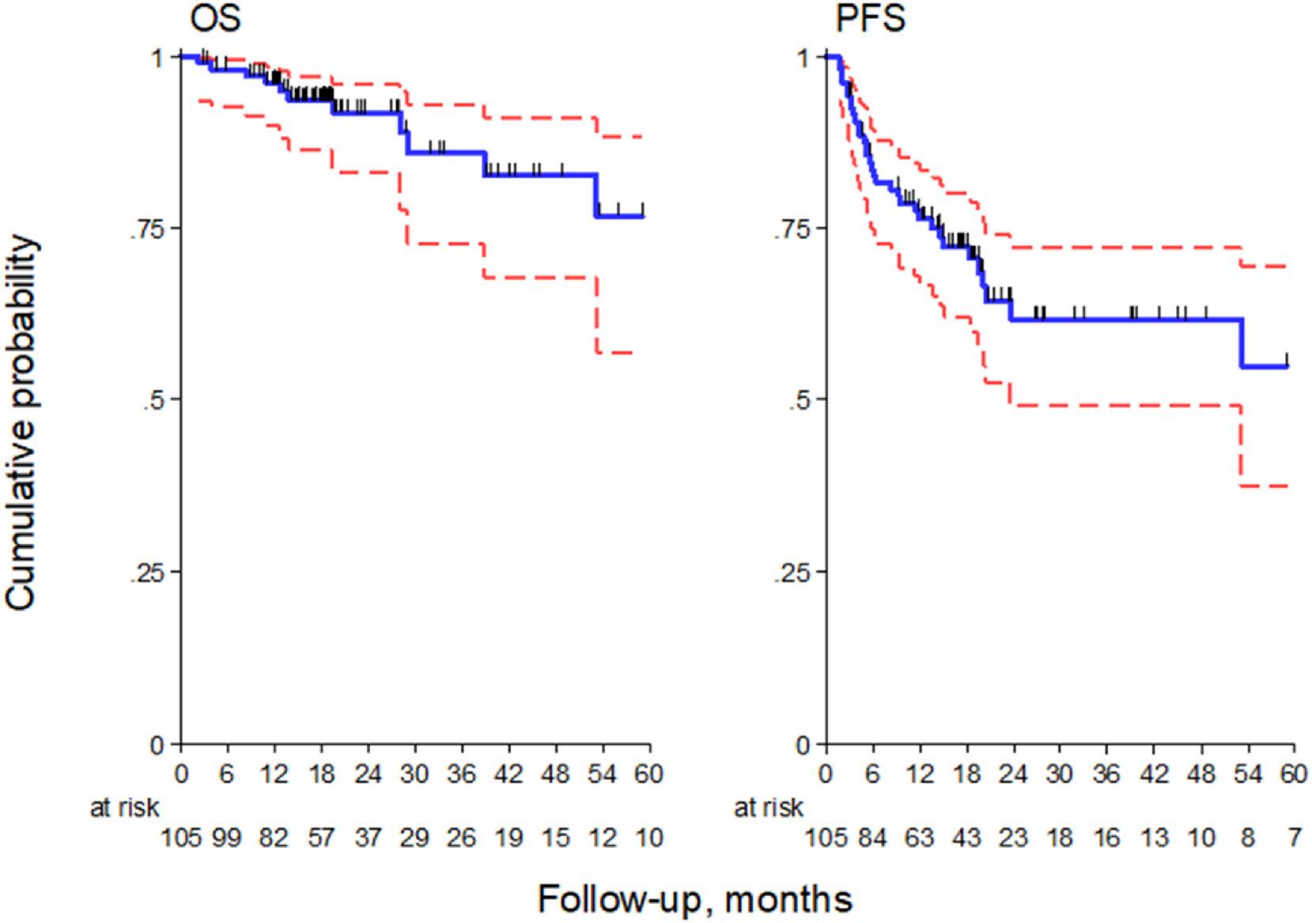
Kaplan–Meier plots showing OS and PFS (red lines represent 95% CI). CI, confidence interval; OS, overall survival; PFS, progression‐free survival

**FIGURE 2:**
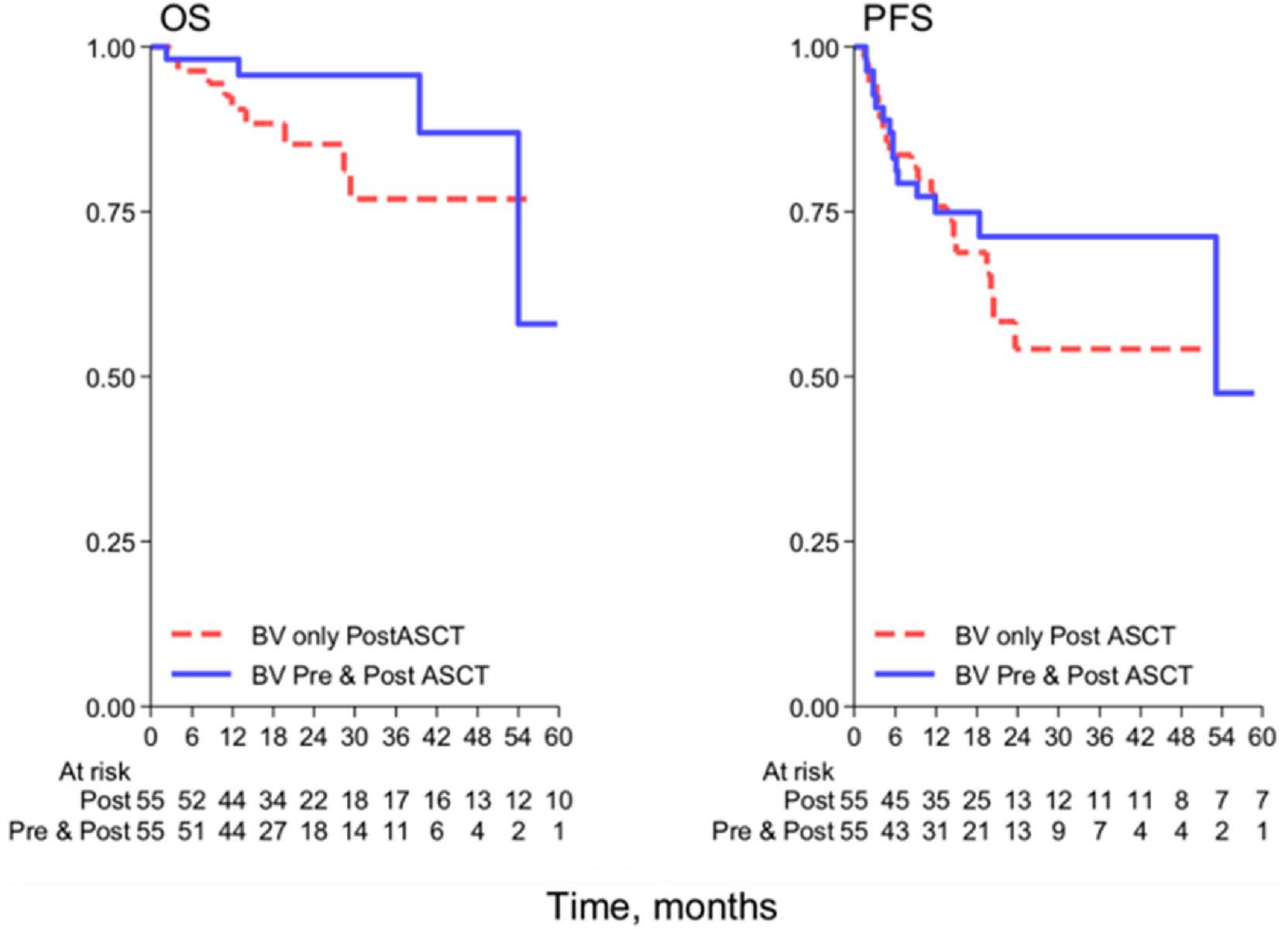
Kaplan–Meier plots showing OS and PFS according to BV exposure. BV, brentuximab vedotin; OS, overall survival; PFS, progression‐free survival

Concerning efficacy data, 3‐year PFS and OS were 62% (95% CI, 49–72) and 86% (95% CI, 73–93), respectively (Figure 1). Median PFS and OS were not reached. The survival analysis according to BV exposure (only post‐ASCT vs. BV also as salvage therapy) showed 3‐year PFS and OS of 54% and 71% (*p* = 0.532), 77% and 96% (*p* = 0.299), respectively (Figure 2).

In the subgroup of 22 patients presenting a DS 4–5 post‐ASCT, we recorded a higher number of conversions to CR during BV consolidation therapy in BV naïve patients (9/15; 60%) than in those already exposed to BV before ASCT (3/7; 43%). Among the whole population receiving maintenance, the median time to best response was 4 months. Table 4 summarizes disease assessments according to the treatment phase. Among the patients achieving CR during BV consolidation (median duration of response [DOR] of 59.7 months), two were referred to allo‐SCT and only one patient presented disease progression. Among PFS events, 30 relapses/progressions and three deaths for any cause were recorded. Only one case of relapse was reported in the subgroup of 17 (16%) patients who did not present any high‐risk factor.

**TABLE 4:** Disease assessments according to treatment phase

Phase	BV pre (54 pts)					BV naïve (51 pts)				
	CR	PR	SD	PD	NA	CR	PR	SD	PD	NA
Pre‐ASCT	40	8	2	4	–	33	8	9	1	–
Post‐ASCT	43	7	2	2	–	33	7	5	6	–
Post‐maintenance	45	3	2	4	–	39	2	3	6	1
Last FUP	44	3	1	4	2	40	3	0	6	2

Abbreviations: ASCT, autologous stem cell transplantation; BV, brentuximab vedotin; CR, complete response; NA, not available; PD, progressive disease; PR, partial response; SD, stable disease.

Concerning relapsing patients, 25 (83%) received subsequent treatment, 12 of whom proceeded to allo‐SCT. No relapses were reported among the group of patients who interrupted the treatment due to toxicity.

A univariable analysis was conducted to evaluate the prognostic role of the main risk factors (Table 5).

**TABLE 5:** Univariable Cox proportional hazards regression analysis in OS and PFS

Factor		OS – HR (95% CI)	*p*	PFS – HR (95% CI)	*p*
Age at BV	<45	1.00		1.00	
	≥45	1.63 (0.43–6.24)	0.476	0.90 (0.39–2.07)	0.796
Sex	F	1.00		1.00	
	M	1.21 (0.36–4.03)	0.755	1.72 (0.84–3.52)	0.141
Stage relapse	I–II	1.00		1.00	
	III–IV	1.01 (0.30–3.35)	0.987	1.63 (0.82–3.24)	0.159
Extranodal relapse	No	1.00		1.00	
	Yes	1.12 (0.29–4.24)	0.869	1.55 (0.74–3.26)	0.249
PET after 1st treat	DS 1–3	1.00		1.00	
	DS 4–5	3.11 (0.78–12.5)	0.109	1.38 (0.65–2.96)	0.402
PET pre‐ASCT	DS 1–3	1.00		1.00	
	DS 4–5	4.71 (1.18–18.9)	0.029	3.81 (1.80–8.09)	<0.001
PET post‐ASCT	DS 1–3	1.00		1.00	
	DS 4–5	2.93 (0.76–11.4)	0.119	3.28 (1.42–7.58)	0.005
AETHERA code	0–1	1.00		1.00	
	2–3	1.09 (0.32–3.64)	0.890	2.35 (1.15–4.78)	0.019
Refractory disease	No	1.00		1.00	
	Yes	2.06 (0.54–7.83)	0.289	3.25 (1.51–7.00)	0.003
Remission, months	12+	1.00		1.00	
	<12	1.04 (0.31–3.44)	0.949	0.94 (0.48–1.87)	0.871
No. lines pre‐BV	0–1	1.00		1.00	
	>1	3.14 (0.96–10.3)	0.059	0.82 (0.34–1.99)	0.662

*Note*: AETHERA code: the presence of pre‐autoSCT risk factors (refractory disease, CR < 12 months, extranodal relapse).

Abbreviations: ASCT, autologous stem cell transplantation; BV, brentuximab vedotin; CI, confidence interval; HR, hazard ratio; OS, overall survival; PFS, progression‐free survival.

The only feature significantly associated with reduced PFS and OS was a DS 4–5 before ASCT (HR 3.81; 95% CI, 1.80–8.09; *p* < 0.001). DS 4–5 after ASCT (HR 3.28; 95% CI, 1.42–7.58; *p* = 0.005), the presence of refractory disease before salvage therapy (HR 3.25; 95% CI, 1.51–7.00; *p* = 0.003) and the presence of two or more high‐risk factors (HR 2.35; 95% CI, 1.15–4.78; *p* = 0.019) were associated with reduced PFS.

The presence of refractory disease (HR 4.46; 95% CI, 1.67–11.9; *p* = 0.003) and a DS 4–5 after ASCT (HR 3.14; 95% CI, 1.41–6.97; *p* = 0.005) were the only features significantly associated with reduced PFS in the multivariable analysis (Table 6).

**TABLE 6:** Multivariable Cox proportional hazards regression analysis in PFS

Factor		PFS – HR (95% CI)	*p*
Refractory disease	No	1.00	0.003
	Yes	4.46 (1.67–11.9)	
PET post‐ASCT	DS 1–3	1.00	
	DS 4–5	3.14 (1.41–6.97)	0.005

Abbreviations: ASCT, autologous stem cell transplantation; HR, hazard ratio; PFS, progression‐free survival.

## 4 DISCUSSION

The use of BV as post‐ASCT consolidation treatment has been validated in the 2015 AETHERA trial, which reported its advantage in terms of PFS compared to the placebo arm.

In our series, after a median follow‐up of 20 months, we recorded an estimated 3‐year PFS of 62%, which we consider comparable to the 2‐ and 5‐year PFS rate of 63% and 59%, respectively, reported in the first and in the extended follow‐up publications of the AETHERA trial.^15^, ^16^

Whether consolidation with BV confers an OS advantage still remains a matter of debate. The first AETHERA publication did not show any differences in OS between the two arms, while its recent update did not analyze the data due to the lack of sufficient events to be able to draw any significant conclusions.

We report an estimated 3‐year OS of 86%, which appears to be similar to the survival curves from the AETHERA trial and which confirms the excellent result achieved with the available salvage therapies in HL.

It is important to underline that, differently from the AETHERA trial, our study included 16% of patients who did not present any high‐risk feature. Nevertheless, our population presented a high frequency of poor prognostic features: 54% had received at least two prior lines of salvage therapy before ASCT and 29% had at least two pre‐ASCT high‐risk features.

Overall, 54% of patients completed the 16 cycles of BV treatment, which is in line with previous reports. It is important to note that almost one third of patients discontinued treatment due to physician decision or to proceed to further consolidation with allo‐SCT. Currently, given the greater access to checkpoint inhibitors and a broader knowledge of how to manage drug‐specific toxicities, it is reasonable to believe that the entire consolidation program could be carried out in a higher proportion of patients.

Of note, we also included 54 patients who received BV before ASCT, mostly due to an incomplete response after salvage therapy that would have limited the success rate of ASCT consolidation. Our study did not permit an assessment of the efficacy of BV pre‐ASCT but was able to investigate the toxicity and efficacy of BV pre‐ASCT compared to that of standard post‐ASCT BV consolidation. Based on our results, administrating BV pre‐ASCT was not associated with any change in the efficacy of standard BV consolidation. Moreover, the safety profile of standard BV consolidation was not affected by prior exposure to the same drug.

Regarding toxicity, we chose to describe non‐hematological events, specifically peripheral neurologic events and their evolution, because of a specific interest in PN associated with BV use. Non‐hematological grade 3–4 AEs were recorded in 11% of cases, representing one third of the causes of treatment discontinuation. Peripheral neuropathy was reported in 21% of cases, mostly grade 1–2, and was the reason for discontinuation of BV in eight cases. Interestingly, almost 60% of the patients did not show any improvement in AE grade. These data are in contrast with what is reported in the literature, where 80%–85% of patients with PN had an improvement or resolution of symptoms after a median of from 9.9 to 23.4 weeks.^14^, ^15^, ^24^ This difference can be explained by the high percentage of grade 1–2 AEs in our series, which may be more difficult to document and downgrade in a retrospective setting.

In conclusion, BV treatment as post‐ASCT consolidation represents an effective, safe option for HL patients even in the real‐life setting. The treatment also seems to be effective in patients already exposed to the drug before ASCT, a condition frequently seen in clinical practice. Further data are needed to evaluate whether the treatment confers an advantage in terms of OS, especially considering new available post‐ASCT treatment.

## CONFLICT OF INTEREST

A. Pulsoni, M. Cantonetti, A. Rossi, V.R. Zilioli, A. Fabbri, F. Merli have a consultant or advisory role for Takeda; M. Cantonetti, A. Visentin, A. Fabbri received educational grant from Takeda.

### PEER REVIEW

The peer review history for this article is available at https://publons.com/publon/10.1002/hon.2939.

## Data Availability

The data that support the findings of this study are available from the corresponding author upon reasonable request.
